# Blood-brain barrier disruption mediates the association between cerebral small vessel disease and clinical outcome after stroke: a secondary analysis of the Lesion Evolution in Stroke and Ischemia on Neuroimaging study

**DOI:** 10.3389/fstro.2024.1510359

**Published:** 2024-12-04

**Authors:** Derrick N. Okine, Kyle C. Kern, Marie Luby, Lawrence L. Latour, Rebecca F. Gottesman

**Affiliations:** ^1^David Geffen School of Medicine, University of California, Los Angeles, Los Angeles, CA, United States; ^2^Intramural Research Program, National Institute of Neurological Disorders and Stroke, National Institutes of Health, Bethesda, MD, United States; ^3^Department of Neurology, David Geffen School of Medicine, University of California, Los Angeles, Los Angeles, CA, United States; ^4^Department of Neurology, West Los Angeles Veterans Affairs Medical Center, Los Angeles, CA, United States

**Keywords:** HARM, white matter hyperintensities, cerebral small vessel disease, ischemic stroke, post-stroke outcome

## Abstract

**Introduction:**

White matter hyperintensities (WMH) in patients presenting with acute ischemic stroke are associated with worse clinical outcomes, but the mechanisms underlying this association are unclear. The purpose of this study was to determine whether blood-brain barrier (BBB) disruption, detected as the hyperintense acute reperfusion marker (HARM) on post-gadolinium follow-up FLAIR MRI, is associated with WMH and mediates the association between WMH and stroke outcomes.

**Methods:**

This is a secondary analysis of the LESION study, where patients with suspected acute ischemic stroke who were candidates for acute stroke intervention or had a baseline NIHSS ≥4 underwent serial multimodal MRI within 24 h of last-known-well time, and again at 2 or 24 h. WMH were visually graded on baseline FLAIR for presence and severity (minor or moderate-severe). HARM was evaluated on post-gadolinium FLAIR for presence and severity (minor, severe focal or severe diffuse). Using binomial and multinomial logistic regression, we tested whether WMH grade was associated with presence or severity of HARM, covarying for demographics, vascular risk factors, and stroke characteristics in sequential models. Finally, we used structural equation models to test the mediation effects of severe HARM on the association between WMH and stroke outcomes, including discharge NIHSS, hemorrhagic transformation, and 90-day modified Rankin scale.

**Results:**

For 213 stroke patients (mean age 70 years, 54% female), higher WMH grade was associated with increased risk for severe diffuse HARM (OR: 3.37, 95% CI: 1.45–7.81), although not after adjusting for vascular risk factors or stroke characteristics. In our univariate model, severe HARM had a partial mediating effect between WMH and discharge NIHSS, explaining 23% of the association.

**Discussion:**

These findings suggest a possible association between severe diffuse HARM and WMH severity. The relationship between WMH severity and early stroke outcome may be mediated by blood-brain barrier disruption.

## 1 Introduction

White matter hyperintensities (WMH) are a manifestation of cerebral small vessel disease associated with age, vascular risk factors (O'Sullivan, 2008), and cognitive decline (Ryu et al., 2017). In patients with acute ischemic stroke, WMH are associated with worse clinical outcome and post-stroke cognitive impairment (O'Sullivan, 2008; Ryu et al., 2017; Kliper et al., 2014; Wardlaw et al., 2009). One proposed mechanism for this association is blood-brain barrier (BBB) disruption.

In the setting of acute ischemic stroke, patients with WMH have been found to have increased BBB leakage in brain tissue (Zhang et al., 2017; Taheri et al., 2011; Topakian et al., 2010), which in turn is associated with cytotoxic edema and hemorrhagic transformation (HT) (Kassner and Merali, 2015; Latour et al., 2004). BBB disruption can be visualized on FLAIR MRI following gadolinium (Gd) contrast administration. Since Gd-based contrast agents do not cross the BBB, and FLAIR typically suppresses the signal from cerebrospinal fluid (CSF), detection of FLAIR hyperintense CSF signal following Gd administration indicates increased BBB disruption (Warach and Latour, 2004). This phenomenon is known as hyperintense acute reperfusion marker (HARM) (Latour et al., 2004; Kidwell et al., 2011). HARM is a predictor for treatment complications such as higher risk of reperfusion injury, HT, and stroke recurrence—all of which may lead to poor stroke outcomes and subsequent cognitive deficits (Derraz et al., 2022; Wouters et al., 2021). Given these findings, it is possible that increased BBB disruption mediates the association between cerebral small vessel disease and worse clinical outcomes after stroke.

We sought to determine whether pre-existing cerebral small vessel disease, as indicated by WMH, would be associated with BBB disruption, shown by HARM on follow-up MRI. We hypothesized that this relationship might be modified in the setting of acute interventions and by different stroke severity [that is, in people with high vs. low National Institutes of Health Stroke Scale (NIHSS)], with stronger associations in individuals with higher NIHSS or those who received an acute intervention. Finally, we evaluated whether HARM mediated the known association between WMH severity and post-stroke clinical outcomes indicated by NIHSS at discharge, modified Rankin Scale (mRS) at 90-days, and HT.

## 2 Methods

### 2.1 Human subjects research approval

The data used in this study consisted of a de-identified, de-linked dataset consisting of clinical and imaging data, and as such, is not considered human subjects research. The dataset was created for purposes of research and deposited in a repository under NIH OHSR#1360 and #4333, with the approval and oversight of the NIH Office of Human Subjects Research protections.

### 2.2 Data availability

Data used for this analysis can be shared upon reasonable request to the corresponding author under a formal data sharing agreement and with approval from the requesting researcher's local ethics committee.

### 2.3 Patient sample

This is a secondary analysis of the Lesion Evolution in Stroke and Ischemia on Neuroimaging (LESION) Study, a study designed to characterize the MRI targets of potentially treatable acute ischemic stroke (Derraz et al., 2022). The LESION study was comprised of acute ischemic stroke patients screened by the National Institute of Neurological Disorders and Stroke (NINDS) Stroke Team that presented to either Suburban Hospital in Bethesda, Maryland (from August 1999 to October 2009) or MedStar Washington Hospital Center in Washington, DC (from September 2004–October 2009). Stroke patients who were considered for acute recanalization therapies or presented with an NIHSS ≥4 and were screened with multimodal MRI within 24 h of last known well time were included (Derraz et al., 2022). This secondary analysis included patients who had sufficient quality MRI at both baseline presentation and at follow-up of 2 and/or 24 h. Patients with unknown last known well time were excluded. For patients with multiple admissions for stroke, only the first qualifying admission was included.

### 2.4 Imaging protocol and analysis

MRI was performed using 1.5T (Twin-speed, General Electric) and 3.0 T (Achieva, Philips) clinical scanners using previously described protocols (Luby et al., 2011; Shah et al., 2015). MRI was acquired at baseline and at 2-h and/or 24-h follow-up time points. MRI sequences included colocalized diffusion-weighted imaging (DWI), gradient echo (GRE), fluid-attenuated inversion recovery (FLAIR), and dynamic susceptibility contrast perfusion-weighted imaging (DSC-PWI). For the DSC-PWI, a single-dose gadolinium contrast injection of 0.1 mmol/kg was administered (Derraz et al., 2022).

Images were assessed for WMH, HARM, and HT by vascular neurologists and experienced imaging scientists who were blinded to acute presentation, interventions, and clinical outcomes (Derraz et al., 2022). WMH were rated qualitatively on baseline pre-contrast FLAIR using a simplified Fazekas scale (Fazekas et al., 1987) with categorizations of absent, minor, and moderate to severe. HARM was evaluated in the same reading sessions on 2 and 24-h follow-up FLAIR for presence and severity, classified as absent, minor, severe focal, or severe diffuse HARM. HARM was classified as “severe focal” when visible on ≥10 slices and “severe diffuse” when present in bilateral hemispheres (Kim et al., 2021). HARM that met criteria for both severe focal and severe diffuse was classified as severe diffuse. For patients with MRI scans at both 2 and 24 h, the earlier timepoint and the corresponding HARM classification was used for analysis to keep the number of gadolinium doses constant (~2, across all patients). HT was rated using the follow-up GRE as hemorrhagic infarction (HI) 1, HI2, parenchymal hematoma (PH) 1, and PH2 using the adapted Heidelberg scale (von Kummer et al., 2015).

### 2.5 Covariates

Demographic factors including age, sex, and race were collected on admission. Comorbid vascular risk factors including hypertension, atrial fibrillation, diabetes, and smoking status were recorded upon hospital presentation by the clinical team or extracted from the medical record by research staff. NIHSS was assessed at presentation, 24-h, and hospital discharge by the clinical team. mRS was assessed through interview at discharge and via telephone at 90 days by the clinical team or a research nurse. Additionally, use of any acute stroke treatments, intravenous alteplase (IV tPA) and/or endovascular therapy (EVT), was also considered as a covariate and separately as an effect modifier.

### 2.6 Statistical analysis

All statistical analyses were performed in Stata/SE v17.0. First, we considered descriptive analyses comparing WMH categories; one-way ANOVA and Fisher's exact tests were used to compare continuous and categorical variables across WMH categories, respectively. For multivariable analyses, the primary independent variable was WMH. For the primary analysis, HARM was the dependent variable. Clinical outcomes included 24-h and discharge NIHSS, mRS at 90 days, and HT. We used binomial logistic regression to determine if WMH presence or severity was associated with HARM severity, classified as any HARM (yes/ no) and subsequently classified as any severe HARM (yes/no; thus, the reference group included individuals with minor HARM but without severe HARM). We then used multinomial regression to test whether WMH presence or severity predicted HARM severity (minor, severe focal, or severe diffuse, as the dependent variable) with no HARM as a reference.

For each regression, we tested 3 models. The first model included demographic covariates: age, sex, and race. The second model added vascular risk factors: hypertension, diabetes, atrial fibrillation, and smoking status. Model 3 incorporated characteristics of the acute stroke: time of MRI for HARM rating (2 or 24 h), acute intervention (none/ IV tPA/ EVT), and initial NIHSS.

### 2.7 Effect modification

We evaluated interactions between WMH and (1) use of an acute intervention; and (2) NIHSS score at baseline (stratified at 10, our sample median), in separate models, on HARM. For both potential effect modifiers, we considered stratified models and formally tested interaction terms in a combined model.

### 2.8 Mediation of the association between WMH and clinical outcomes by severe HARM

**Figure 1:**
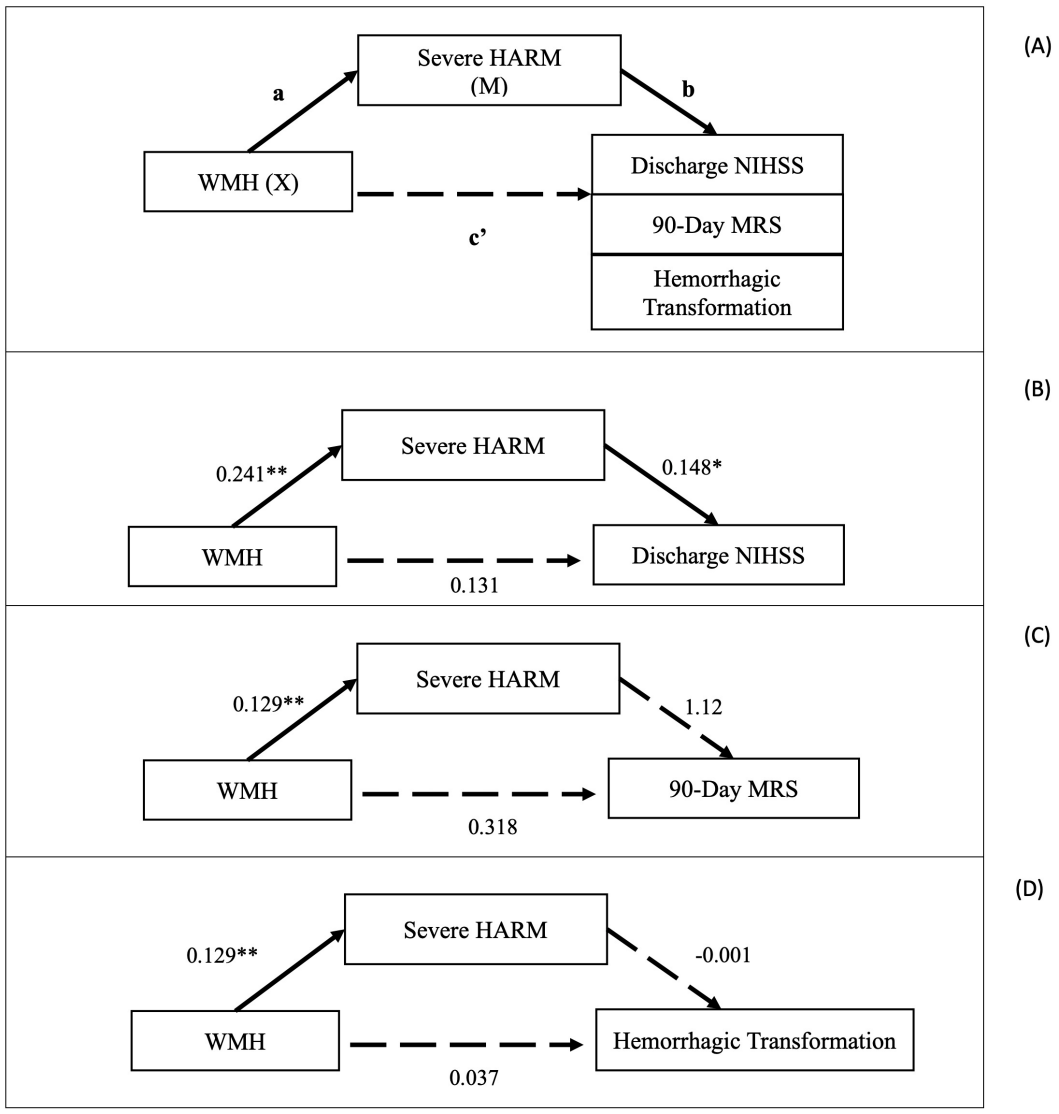
**(A)** Illustration of mediation model where presence of any severe HARM (severe focal, severe diffuse, combined severe focal and severe diffuse) mediates association between severity of WMH and Discharge NIHSS score or 90-Day mRS. **(B)** Unadjusted mediation results for association between white matter hyperintensities and discharge NIHSS as mediated by severe blood-brain barrier disruption (severe HARM). Path coefficients (standardized βs) represented for each association. Asterisks indicate significant associations (*p < 0.05, **p < 0.01). **(C)** Generalized structural equation model for association between white matter hyperintensities and Modified Rankin Scale at 90 days as mediated by severe blood-brain barrier disruption (severe HARM; Unadjusted). Path coefficients (βs) represented for each association. Asterisks indicate significant associations (*p < 0.05, **p < 0.01). **(D)** Generalized structural equation model for association between white matter hyperintensities and hemorrhagic transformation (any vs. none) as mediated by severe blood-brain barrier disruption (severe HARM; Unadjusted). Path coefficients (βs) represented for each association. Asterisks indicate significant associations (*p < 0.05, **p < 0.01). WMH, white matter hyperintensities; HARM, hyperintense acute reperfusion marker; NIHSS, National Institutes of Health Stroke Scale; mRS, Modified Rankin Scale.

To evaluate the presence of severe HARM as a mediator of the relationship between WMH and post-stroke clinical outcome, we used the Baron and Kenny (1986) mediation method. Using structural equation modeling, we tested whether presence of severe HARM mediated associations between WMH severity and each clinical outcome (discharge NIHSS, 90-day mRS or HT) (MacKinnon et al., 2002). For each mediation model, we estimated the amount of the variance of the clinical outcome measure that was explained through severe HARM (indirect effect). We report this estimation as a proportion of the amount explained by the entire mediation model as described by Baron and Kenny (Baron and Kenny, 1986; MacKinnon et al., 2002). Figure 1A illustrates the hypothesized mediation model.

For mediation models, we reported regression coefficients and significance for each independent pathway as well as estimates of the direct causal pathways between WMH and discharge NIHSS, WMH and 90-day mRS, and WMH and HT. All mediations were tested using univariable and multivariable (adjusted for demographics) regressions. These mediation analyses were evaluated using structural equation modeling for NIHSS at discharge and generalized structural equation modeling for HT and 90-day mRS models.

## 3 Results

### 3.1 Patient demographics

**Figure 2:**
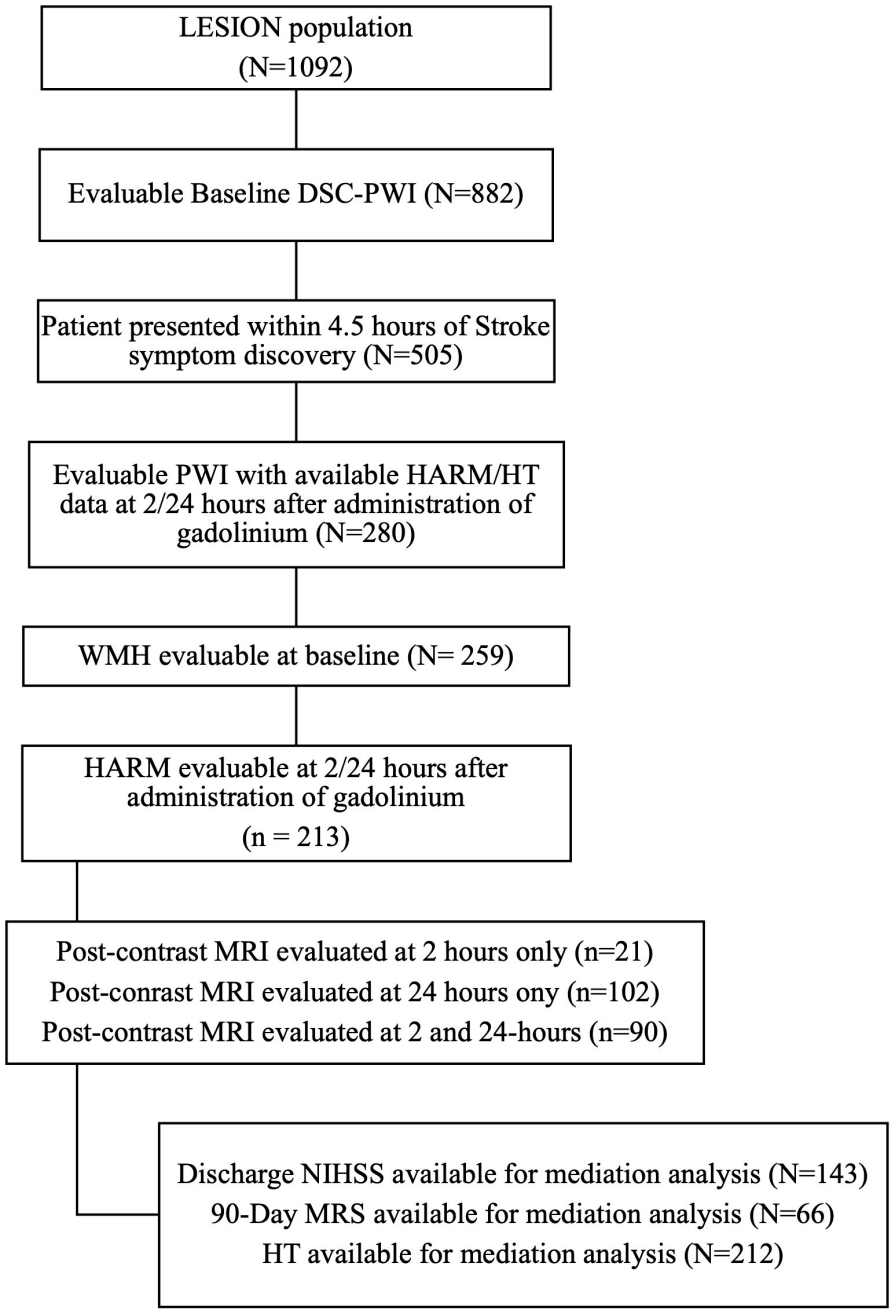
WMH and HARM study inclusion criteria and subgroup analysis characteristics. WMH, white matter hyperintensities; HARM, hyperintense acute reperfusion marker; NIHSS, National Institutes of Health Stroke Scale; HT, hemorrhagic transformation; mRS, modified Rankin Scale; DSC-PWI, dynamic susceptibility contrast perfusion-weighted imaging.

Of 213 patients meeting our inclusion criteria (Figure 2), 100 (47%) had no WMH at baseline, 79 (37%) had minor, and 34 (16%) had moderate to severe WMH. Older age and hypertension were each associated with greater WMH severity, and women were overrepresented in the moderate to severe WMH group (*p* = 0.001; Table 1).

**Table 1:** Characteristics of analytic sample by baseline WMH category (*N* = 213).

	**No WMH (*N* = 100)**	**Minor WMH (*N* = 79)**	**Moderate to severe WMH (*N* = 34)**	*p*-value^*^
Age (year): mean (SD)	62.6 (14.8)	76.6 (11.5)	79.4 (9.7)	< 0.0001
Female sex	52 (52%)	40 (51%)	22 (65%)	0.361
**Race**				0.761
Black	24 (24%)	23 (29%)	9 (26%)	
White	69 (69%)	52 (66%)	22 (65%)	
Asian	5 (5%)	4 (5%)	1 (3%)	
Other	2 (2%)	0	2 (6%)	
**Vascular risk factors**				
Hypertension	63 (63%)	61 (77%)	32 (94%)	0.001
Diabetes	19 (19%)	25 (32%)	10 (29%)	0.129
Current smoking	16 (16%)	9 (11%)	5 (15%)	0.672
**HARM**				0.004
None	45 (45%)	25 (32%)	12 (35%)	
Minor	36 (36%)	28 (35%)	7 (21%)	
Severe focal	15 (15%)	12 (15%)	5 (15%)	
Severe diffuse	4 (4%)	14 (18%)	10 (29%)	
**Intervention**				0.180
None	18 (18%)	16 (20%)	10 (19%)	
IV-tPA	72 (72%)	52 (66%)	22 (65%)	
EVT	6 (6%)	11 (14%)	2 (6%)	
Combination	4 (4%)	0	0	
**Admit NIHSS**				
NIHSS ≥10	49 (49%)	44 (56%)	24 (71 %)	0.094
Mean (SD)	10.51 (7.94)	13.37 (8.41)	14.59 (9.58)	0.017
Range	0–32	2–37	1–40	
MRS at 90 days: mean (SD)	1.94 (1.92)	3 (1.98)	2.44 (2.30)	0.152
Discharge NIHSS: mean (SD)	7.45 (12.37)	14 (15.66)	12 (15.37)	0.036
**HT**				0.737
None	70 (71%)	51 (65%)	22 (65%)	
HI 1	12 (12%)	18 (23%)	6 (17%)	
HI 2	9 (9%)	5 (6%)	3 (9%)	
PH 1	3 (3%)	3 (4%)	1 (3%)	
PH 2	5 (5%)	2 (2%)	2 (6%)	

N (%) unless otherwise specified.

HARM, hyperintense acute reperfusion marker; WMH, white matter hyperintensities; NIHSS, NIH Stroke Scale; IV-tPA, intravenous tissue plasminogen activator (alteplase); EVT, endovascular thrombectomy; MRS, Modified Rankin Scale; HT, worst rating of hemorrhagic transformation (as shown by Heidelberg ECASS II classification at 2/24-h time window after baseline presentation).

^*^p-values calculated using Fisher's exact test (categorical variables) or one-way ANOVA (continuous variables).

Most patients were treated with IV-tPA (146, 69%), reflecting the inclusion criteria for our study. A minority of the patients in the analytic sample had no HARM present (Table 1; 82, 38%).

### 3.2 Presence and categorization of WMH and presence of HARM

Patients with WMH at baseline did not have an increased risk of any HARM on follow-up MRI regardless of WMH categorization. However, in unadjusted models, the presence of any (vs. no) WMH was specifically associated with an elevated risk of severe HARM (combining severe focal, severe diffuse, and a combination of focal and diffuse; Table 2; OR 2.43, 95% CI 1.29–4.56). Compared to individuals with no WMH, minor WMH showed an elevated risk (Table 2; OR 2.09, 95% CI 1.05–4.15), while moderate to severe WMH was associated with the highest risk of severe HARM (Table 2; OR 3.37, 95% CI 1.45–7.81). This association was no longer statistically significant when demographics, vascular risk, and characteristics of the stroke were included as covariates in sequential models (Table 2).

**Table 2:** Associations (odds ratios) between white matter hyperintensity grade and presence of any HARM (mild, severe focal, or severe diffuse HARM) or severe HARM (severe focal or severe diffuse HARM).

	**Unadjusted (95% CI)**	***p***	**Model 1 (95% CI)**	***p***	**Model 2 (95% CI)**	***p***	**Model 3 (95% CI)**	***p***
**WMH and presence of HARM**								
Any white matter hyperintensities (yes/no)	1.68 (0.96, 2.93)	0.067	1.40 (0.72, 2.72)	0.321	1.25 (0.63, 2.49)	0.525	1.30 (0.64, 2.66)	0.468
**Categories of WMH and presence of HARM**								
None	1 (ref)		1 (ref)		1 (ref)		1 (ref)	
Minor	1.77 (0.95, 3.27)	0.070	1.50 (0.74, 3.04)	0.266	1.39 (0.67, 2.90)	0.381	1.39 (0.65, 2.98)	0.395
Moderate to severe	1.5 (0.67, 3.36)	0.324	1.12 (0.48, 2.91)	0.717	0.95 (0.37, 2.41)	0.907	1.10 (0.42, 2.88)	0.841
**WMH and presence of severe HARM**								
Any white matter hyperintensities (yes/no)	**2.43 (1.29, 4.56)**	**0.006**	1.72 (0.83, 3.58)	0.145	1.49 (0.71, 3.15)	0.296	1.49 (0.69, 3.21)	0.315
**Categories of WMH and presence of severe HARM**								
None	1 (ref)		1 (ref)		1 (ref)		1 (ref)	
Minor	**2.09 (1.05, 4.15)**	**0.035**	1.51 (0.70, 3.29)	0.297	1.37 (0.62, 3.03)	0.431	1.32 (0.58, 3.00)	0.506
Moderate to severe	**3.37 (1.45, 7.81)**	**0.005**	2.35 (0.92, 5.98)	0.074	1.81 (0.69, 4.74)	0.224	1.95 (0.73, 5.23)	0.185

Model 1: age, sex, race.

Model 2: model 1+ hypertension, diabetes, smoking status, atrial fibrillation.

Model 3: model 2+ time of follow-up MRI (2 or 24 h), intervention, NIHSS.

WMH, white matter hyperintensities; HARM, hyperintense acute reperfusion marker; NIHSS, National Institutes of Health Stroke Scale.

Associations where mediation is found to be statistically significant at the *p* < 0.05 level are indicated in bold.

### 3.3 Presence and categorization of WMH and severity of HARM

**Figure 3:**
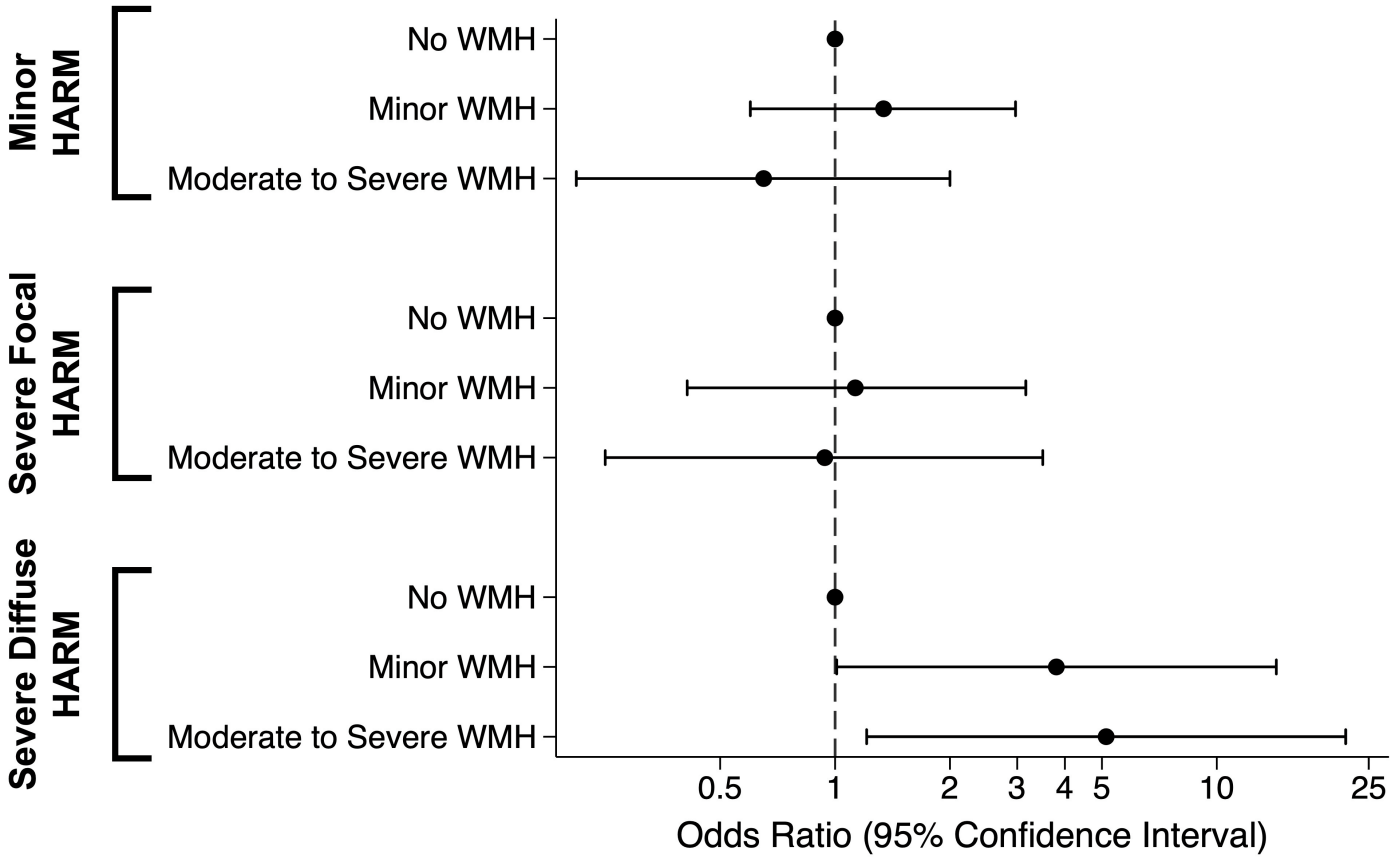
Associations between white matter hyperintensities and severity of HARM in LESION Population, compared to individuals without any HARM. Odds ratios shown are from demographics-adjusted model with age, sex, and race as covariates. WMH, white matter hyperintensities; HARM, hyperintense acute reperfusion marker.

Individuals with any WMH present at baseline had a significantly higher risk of severe diffuse HARM (vs. no HARM) in our demographics-adjusted model (Table 3; OR 4.25, 95% CI 1.21–15.01). When WMH was categorized, and compared to individuals with no WMH, individuals with both minor and moderate to severe WMH had a significantly higher risk of severe diffuse HARM, with the highest risk associated with moderate to severe WMH (Table 3; Model 1 OR 5.13, 95% CI 1.21–21.76). These results were no longer significant once we adjusted for vascular risk factors and characteristics of the stroke (Figure 3).

**Table 3:** Associations (relative risk ratios) between white matter hyperintensity grade and severity of HARM.

	**Unadjusted (95% CI)**	***p***	**Model 1 (95% CI)**	***p***	**Model 2 (95%CI)**	***p***	**Model 3 (95% CI)**	***p***
**Relative risk ratios for HARM for patients with any WMH present**								
Minor HARM	1.18 (0.63, 2.24)	0.606	1.12 (0.53, 2.38)	0.760	1.05 (0.48, 2.28)	0.910	1.10 (0.49, 2.47)	0.820
Severe focal HARM	1.38 (0.61, 3.13)	0.443	1.07 (0.41, 2.80)	0.885	0.91 (0.34, 2.42)	0.846	0.89 (0.32, 2.49)	0.822
Severe diffuse HARM	**7.30 (2.32, 22.92)**	**0.001**	**4.25 (1.21, 15.01)**	**0.024**	3.54 (0.98, 12.72)	0.053	3.37 (0.91, 12.42)	0.068
**Relative risk ratios for HARM for patients with minor WMH**								
Minor HARM	1.4 (0.70, 2.80)	0.343	1.34 (0.60, 2.97)	0.473	1.28 (0.56, 2.91)	0.562	1.30 (0.55, 3.06)	0.545
Severe focal HARM	1.44 (0.58, 3.33)	0.429	1.13 (0.41, 3.16)	0.813	1.01 (0.36, 2.89)	0.972	0.92 (0.31, 2.77)	0.885
Severe diffuse HARM	**6.30 (1.88, 21.21)**	**0.003**	**3.80 (1.01, 14.32)**	**0.049**	3.36 (0.87, 12.90)	0.078	3.16 (0.80, 12.51)	0.102
**Relative risk ratios for HARM for patients with moderate-severe WMH**								
Minor HARM	0.73 (0.26, 2.04)	0.548	0.65 (0.21, 2.00)	0.453	0.55 (0.17, 1.76)	0.315	0.64 (0.19, 2.09)	0.457
Severe focal HARM	1.25 (0.38, 4.13)	0.715	0.94 (0.25, 3.50)	0.251	0.66 (0.17, 2.57)	0.549	0.82 (0.20, 3.33)	0.777
Severe diffuse HARM	**9.37 (2.50, 35.20)**	**0.001**	**5.13 (1.21, 21.76)**	**0.026**	3.65 (0.84, 15.94)	0.085	3.61 (0.80, 16.24)	0.094

All reported RRRs relative to no WMH [reference group = OR/RRR (1) for each].

Model 1: age, sex, race.

Model 2: model 1+ hypertension, diabetes, smoking status, atrial fibrillation.

Model 3: model 2+ time of MRI (for HARM rating), intervention, NIHSS.

WMH, white matter hyperintensities; HARM, hyperintense acute reperfusion marker; NIHSS, National Institutes of Health Stroke Scale.

Associations where mediation is found to be statistically significant at the *p* < 0.05 level are indicated in bold.

### 3.4 Effect modification by acute intervention and NIHSS

To determine whether the association between WMH and HARM is influenced by acute treatment or stroke severity (presenting NIHSS < 10 vs. ≥10), we tested for interaction effects. Although we acknowledge small numbers in these subgroups, neither acute intervention nor presenting NIHSS modified the association between WMH and presence of HARM (results not shown).

### 3.5 WMH and clinical outcomes as mediated by severe HARM

The mediation analysis for discharge NIHSS included 143 patients within the analytic sample who had discharge NIHSS scores, WMH, and HARM measured. Steps 1–3 outlined by the Baron and Kenny method (see Section 2) were satisfied for this analysis. WMH were independently associated with both discharge NIHSS (step 1: β, 3.42; 95% CI, 0.13–6.72; *p* = 0.042) and severe HARM (step 2: β, 1.87; 95% CI, 1.24, 2.81; *p* = 0.003). Severe HARM was also associated with discharge NIHSS (step 3: β, 6.42; 95% CI, 1.07–11.8; *p* = 0.019).

For the unadjusted mediation, severe HARM partially mediated the association between WMH and discharge NIHSS, explaining 23% of the association (Figure 1). Once this model was adjusted for age, sex, and race, the mediation effect was no longer significant, but the proportion of the total model explained (nonsignificantly) via severe HARM was 64%.

Using the same methods, we evaluated whether severe HARM mediated the relationship between WMH and 90-Day mRS for 66 participants who had 90-day follow-up data. Neither univariable nor multivariable adjusted models passed steps 1 to 3 for further evaluation of a Baron and Kenny mediation, likely due to the small sample size. WMH were not independently associated with mRS at 90 days (step 1: β, 0.46; 95% CI, −0.23, 1.15; *p* = 0.188). However, WMH were associated with severe HARM (step 2: β, 1.87; 95% CI, 1.24, 2.81; *p* = 0.003), and severe HARM was associated with 90-Day mRS (step 3: β, 1.24; 95% CI, 0.06, 2.43; *p* = 0.040) in this subgroup.

We found similar results when evaluating how severe HARM mediates the association between WMH and HT (*n* = 212 evaluated for HT outcome). WMH were not independently associated with the presence of any HT, but, as in the above analyses, they were associated with severe HARM. However, severe HARM was not associated with the presence of any HT (Table 4). In this mediation model, severe HARM did not contribute to the association between WMH and the presence of any HT.

**Table 4:** Findings for mediation analysis of discharge NIHSS (*N* = 143) and 90-day MRS (*N* = 66).

**Association**	**Beta coefficients**	**Significance**
**Mediation of the association between WMH and discharge NIHSS by severe HARM**		
WMH–severe HARM	0.148	**0.003**
Severe HARM–discharge NIHSS	5.386	**0.0496**
WMH–discharge NIHSS total	2.627	0.12
Indirect effect = 0.797		
Total effect = 3.423		
Percent of association between WMH and discharge NIHSS mediated by presence of severe HARM (indirect/total effect) = **23% significant partial mediation**		
**Mediation of the association between WMH and MRS at 90 days by severe HARM**		
WMH–severe HARM	0.129	**0.002**
Severe HARM-−90 days MRS	1.118	0.061
WMH–90 days MRS	0.318	0.352
Indirect effect = 0.144		
Total effect = 0.462		
Percent of association between WMH and 90 days MRS mediated by severe HARM (indirect/total effect) = 31% (nonsignificant)		
**Mediation of the association between WMH and Hemorrhagic transformation (any vs. none) by severe HARM**		
WMH–severe HARM	0.129	**0.002**
Severe HARM–HT	−0.001	0.985
WMH–HT	0.037	0.411
Indirect effect = 0.000		
Total effect = 0.037		
Percent of association between WMH and HT PH2 mediated by severe HARM (indirect/total effect) = 0%		

WMH, white matter hyperintensities; HARM, hyperintense acute reperfusion marker; mRS, Modified Rankin Scale; NIHSS, National Institutes of Health Stroke Scale.

Associations where mediation is found to be statistically significant at the *p* < 0.05 level are indicated in bold.

## 4 Discussion

In this sample of acute ischemic stroke patients, we find that blood-brain barrier disruption, as measured by HARM, is associated with WMH, and partially mediates the association between WMH and discharge NIHSS. While the presence of WMH, regardless of severity, is associated with increased risk for severe HARM, the highest risk was found in those with moderate to severe WMH. This graded response indicates that the severity of SVD burden relates to the presence of extensive blood-brain barrier disruption during acute ischemic stroke. While the observed partial mediation loses significance once we adjust for demographics, our univariate findings suggest a potential underlying role for acute BBB disruption in early stroke outcomes.

These findings are consistent with other studies that have shown that a greater pre-stroke burden of WMH is associated with worse stroke and clinical outcomes (Zhang et al., 2017; Topakian et al., 2010; Li et al., 2017; Freeze et al., 2020; Arba et al., 2017). Our findings implicate greater BBB disruption as a potential mechanism for this association. Increased inflammation and BBB disruption during the acute stroke period might contribute to reperfusion injury, more severe infarction, or lead to early complications such as post-stroke delirium, thereby impeding early recovery. In clinical studies, the degree of BBB disruption is associated with risk for HT and worse stroke outcomes (Bang et al., 2007; Nadareishvili et al., 2019). As such, severe BBB disruption is considered deleterious in acute ischemic stroke. Identifying predictors and treatments for BBB disruption may help advance stroke therapy.

In the current study, we show that even with a qualitative grading of WMH, there is an association between SVD burden and BBB disruption in acute ischemic stroke patients. Although this finding is present in our demographics-adjusted model, further adjustment for vascular risk factors and acute stroke characteristics led to loss of significance. WMH, an imaging biomarker for cerebral small vessel disease, are thought to reflect the cumulative burden of cerebrovascular risk factors and aging. However, the loss of significance with inclusion of these additional vascular risk factors suggests that there may be additional vascular-related drivers for increased BBB permeability which are primarily responsible for the observed association, and that WMH is simply one marker of this underlying accumulated vascular risk. The use of a continuous measure of WMH volume or a composite measure of multiple MRI markers of SVD (Duering et al., 2023) may yield stronger associations. While each of the vascular risk factors considered in Model 2 are associated with worse stroke outcomes, Diabetes Mellitus type 2 is also associated with increased BBB permeability in humans without stroke (Starr et al., 2003), and hypertension increases BBB disruption in pre-clinical models (Setiadi et al., 2018). We hypothesize that the accumulated vascular injury from aging and acquired risk factors contributes to increased BBB permeability, but we cannot exclude a potential contribution from inherited factors. In previous studies of participants with inherited SVD without acute stroke, BBB changes were heterogeneous (Ying et al., 2024; Walsh et al., 2021).

While BBB permeability during acute stroke is related to chronic SVD white matter injury, we predicted that acute stroke characteristics such as stroke severity would also modify the WMH relationship with HARM. Prior studies have shown that increased stroke severity is associated with BBB disruption (Latour et al., 2004; Desilles et al., 2013). However, using an NIHSS cutoff of 10, we did not find that stroke severity moderated the effect of WMH on severe HARM. It is possible that the association between WMH and severe HARM may be confounded by other unmeasured contributors, such as the duration of ischemia, infarct volume, successful reperfusion (Latour et al., 2004), stroke etiology (Choi et al., 2017), or potentially gadolinium clearance, or may have been primarily limited due to insufficient power due to small numbers. However, our findings suggest that stroke severity doesn't greatly influence the association between pre-stroke brain health and HARM.

In addition to stroke severity, we hypothesized that the relationship between WMH and HARM might be modified in the setting of acute interventions. We posited that those receiving acute recanalization treatments would have a stronger association between WMH and HARM, but we did not find any effect of intervention. This sample was comprised mostly of IV-tPA patients (69%) due to the inclusion criteria for the original LESION study, with relatively few undergoing endovascular therapy (9%). Thus, sample sizes are small for these stratified analyses so lack of evidence of effect modification may simply be due to inadequate power. Prior studies have shown that direct mechanical manipulation of the vessels is associated with greater blood-brain barrier disruption (Desilles et al., 2013; Luby et al., 2019). However, it is not clear whether endovascular intervention would modify the association between WMH and HARM, as our numbers were too small to evaluate this specific intervention.

Given the known associations between WMH and clinical outcome and our observed association between WMH and HARM, we tested whether HARM plays a mediating role between WMH and post-stroke clinical outcome and might be a potential mechanism by which WMH contributes to poor outcome. In univariate analyses, we did find supportive evidence of a significant partial mediation by severe HARM for the association between WMH and NIHSS at discharge. When we considered 90-day mRS or HT as alternative clinical outcomes, we did not find a total effect of WMH, nor a mediation effect by severe HARM. However, the mRS analysis only included 66 patients with 90-day follow-up data, so this sample size is likely too small for a meaningful mediation analysis (Fritz and MacKinnon, 2007). Additionally, it is possible that the mediatory role of severe BBB disruption may be dwarfed by the stronger influence of demographic (Acton et al., 2022) and clinical factors such as the extent and intensity of rehabilitation therapies, support at home, and interval medical complications.

### 4.1 Limitations

Due to the observational, qualitative nature of this study, there are limitations with our analysis. The use of HARM scores from the first available MRI after baseline may have caused a bias toward lower grades of HARM as extravasation of gadolinium may not have developed to its fullest extent during the earlier window. While early HARM may ultimately reflect more severe BBB disruption long-term, HARM at later timepoints may also reflect impaired clearance mechanisms at the level of the glymphatics or the kidneys. Several potential contributors to blood-brain barrier disruption during acute stroke were not considered in this analysis, including hyperglycemia, revascularization status, or acute blood pressure management. Each of these factors are related to stroke outcome, but their relationship to the presence of HARM should be investigated in future studies. Furthermore, the qualitative scale used for evaluating WMH is based on the Fazekas scale but is a crude measurement that may not comprehensively capture the continuum of SVD changes, nor does it allow evaluation of regional associations between HARM and SVD. An automatic WMH segmentation algorithm using machine learning could provide a more objective, continuous, WMH volume measure. However, established algorithms are challenging to apply to acute stroke MRI protocols that are designed for efficiency. WMH volume segmentation may be confounded by motion or acute and chronic infarcts and may still require manual intervention. Similarly, a more objective machine-learning approach to identifying and classifying HARM would be advantageous for future studies. Such an approach may prove clinically useful for distinguishing HARM from subarachnoid blood, a common dilemma in management of stroke patients immediately post-thrombectomy.

Additionally, even though telephone assessment of mRS is validated and reliable, it remains a subjective patient-derived measure (Janssen et al., 2010). Also, the extent of missing data limits interpretations from this analysis of 90-day outcomes, since the missingness may not be random. Since this model relies on independent contributions of several biological processes, there is a likelihood that factors not captured here such as kidney function or other vascular risk factors could play a role in the hypothesized mediation model (Figure 1A) or confound the association between WMH and HARM. The fact that some of our findings are no longer statistically significant when additional confounders are considered, in sequential models, further supports the likelihood that observed relationships may be confounded. We also note that given the modest sample size, this may also reflect insufficient power to support the hypothesized associations. Even with these limitations, this study informs our knowledge of how small vessel disease, the BBB, and clinical outcomes are associated.

## 5 Conclusions

In a secondary analysis of acute ischemic stroke patients undergoing serial MRI, we demonstrated that WMH and HARM are associated with stroke outcome. Here, we found that the presence of WMH is associated with the risk of severe BBB disruption (HARM), and that HARM partially mediates the association between WMH and early stroke outcome. While increased BBB permeability may reflect the neuroinflammatory response to stroke, underlying SVD may amplify this response, exacerbating stroke injury. Further work is needed to better understand how cerebral small vessel disease contributes to BBB disruption and clinical outcomes in acute ischemic stroke.

## Data Availability

The data analyzed in this study is subject to the following licenses/restrictions. Summary data can be provided upon reasonable request (listed in the paper). Requests to access these datasets should be directed to: Rebecca Gottesman, rebecca.gottesman@nih.gov.
